# Macrophage-myofibroblast transition as a novel therapeutic target in shoulder stiffness: multi-omics study and experimental validation

**DOI:** 10.3389/fcell.2025.1727624

**Published:** 2026-01-06

**Authors:** Beijie Qi, Weihan Yu, Hanyi Wang, Yuqi Li, Shuang Deng, Chengqing Yi

**Affiliations:** 1 Department of Orthopedics, Shanghai Pudong Hospital, Fudan University Pudong Medical Center, Shanghai, China; 2 Department of Sports Medicine, Shanghai General Hospital, Shanghai Jiao Tong University School of Medicine, Shanghai Jiao Tong University, Shanghai, China

**Keywords:** fibrosis, macrophage-myofibroblast transition, multi-omics, periostin, shoulder stiffness

## Abstract

**Background:**

Shoulder stiffness (SS) is a fibrotic disease with pain and reduced range of motion (ROM). The pathogenesis of SS remains unclear. Recent studies reveled that macrophage-myofibroblast transition (MMT) is an important mechanism underlying fibrogenesis, but whether MMT was involved in SS progression remained unknown. This study aimed to clarify the role of MMT in SS pathogenesis, and to evaluate the efficacy of MMT-targeted therapy.

**Methods:**

Shoulder capsules from SS patients were collected, and the mouse SS model was established. Western blot and immunofluorescence were utilized to detect protein expression. Multi-omics analysis was performed in order to identify the potential pathogenic factor. Histological and biomechanical analysis was conducted for the *in vivo* experiments.

**Results:**

Significant capsule fibrosis and ROM restriction were observed in both SS patients and SS mice. Upregulated MMT was detected in SS capsules. Multi-omics analysis identified periostin (POSTN) as the potential pathogenic factor. MMT was induced by POSTN *in vitro*. POSTN knockdown effectively attenuated MMT in mouse SS models, ameliorating capsule fibrosis and improving ROM.

**Conclusion:**

In this study, we proved that MMT was involved in SS progression, and identified POSTN as the key regulator of MMT. POSTN knockdown effectively suppressed MMT, alleviated fibrosis, and restored ROM *in vivo*. This research elucidated a novel mechanism in SS pathogenesis and developed POSTN as a promising therapeutic target for SS.

## Introduction

Shoulder stiffness (SS) is a common fibrotic disease characterized by pain and restricted range of motion (ROM) (Millar et al., 2022). The prevalence of SS is approximately 2%–5%. Diabetes, thyroid diseases, shoulder trauma, and postoperative immobilization are risk factors for SS (Walker-Bone et al., 2004; Neviaser and Neviaser, 1987; Sarasua et al., 2021). The patients suffer from pain and limited ROM, with external rotation mostly restricted (Hand et al., 2007).

Although SS is self-limiting disease, there are still many patients cannot fully recover (Kim et al., 2020). The pathogenesis of SS remains unclear, and the conventional therapy including non-steroidal anti-inflammatory drug, glucocorticoid injection, and physical therapy can only alleviate symptoms but fail to prevent the progression of joint stiffness. Severe SS still requires surgical intervention (Cho et al., 2019; Rangan et al., 2020; Challoumas et al., 2020). Therefore, it is essential to understand the SS pathogenesis for developing therapeutic strategies.

The pathological manifestations of SS mainly include chronic inflammation and capsule fibrosis (Dias et al., 2005). In an inflammatory environment, cytokines such as TGF-β and IL-1 accumulate in the capsule (Chen et al., 2017; Alghamdi et al., 2023). These cytokines recruit and activate fibroblasts to differentiate into myofibroblasts which excessively secret collagen into extracellular matrix (ECM), resulting in capsule fibrosis (Qi et al., 2025a; Luo et al., 2022). Thus, inhibiting the activity of myofibroblasts is the key to ameliorate capsule fibrosis.

In the early stage of SS, the joint capsule is infiltrated by abundant inflammatory cells, including macrophages and mast cells (Ng et al., 2024; Itoi et al., 2016). Recent studies have revealed that macrophages possess highly complex function in inflammatory response (Cao et al., 2025; Wang et al., 2024). Beyond classic M1/M2 polarization, they may transdifferentiate into ​​α-SMA (+) myofibroblasts, and directly contribute to fibrogenesis (Ji et al., 2023; Tang et al., 2021; Ban et al., 2024). This process is called ​​macrophage-myofibroblast transition (MMT) which is a critical source of myofibroblasts in various diseases (Tang et al., 2022; Tang et al., 2020; Wang et al., 2017; Abu El-Asrar et al., 2023; Xiang et al., 2025).

The MMT was first discovered in 2014 by Nikolic-Paterson who revealed that bone marrow-derived macrophages (BMDMs) could transdifferentiate into myofibroblasts, causing renal fibrosis (Nikolic-Paterson et al., 2011). So far, MMT has been observed in various fibrotic diseases and tumor microenvironments (Peng et al., 2025; Tang et al., 2024). Zhuang et al. found that MMT contributed to cardiac fibrosis through m6A modification of IL-11 mRNA (Zhuang et al., 2024). Our previous work also found that MMT contributed to skeletal muscle fibrosis in acute skeletal muscle injury repair (Qi et al., 2024). These findings suggest that ​**​**MMT may possess pan-tissue universality**​**​. While MMT has been increasingly recognized as a critical contributor to fibrosis in multiple tissues, its role in SS remains unexplored. To date, no direct evidence has proved whether MMT occurs in SS progression.

Multi-omics technology​​ is pivotal for decoding the complex networks underlying disease pathogenesis (Ding et al., 2022; Goncalves et al., 2022; Chen et al., 2024; Zhou et al., 2025). It has been widely applied in the investigation of pathogenesis of diverse diseases (Vistain and Tay, 2021; Legut, 2023; Kang et al., 2023; Jin C. et al., 2024). Therefore, this study aims to identify the existence of MMT in SS progression and elucidate the mechanism of MMT-mediated fibrogenesis using multi-omics technology.

## Materials and methods

### Ethical compliance

This study was approved by the Ethics Committee (2025-R-G-059) and the Institutional Animal Care and Use Committee (2025-D-G-083) of Shanghai Pudong Hospital. Written informed consent was obtained from patients for capsules collection.

### Capsule sample collection

Patients were stratified into SS and non-SS (NC) groups based on published criteria (Chen et al., 2010), wherein SS was defined as >25% reduction in passive ROM (flexion, external rotation, and internal rotation) compared to the contralateral side (Sun et al., 2018). Capsules of NC group were collected from patients with shoulder joint instability.

### Establishment, intervention, and biomechanical analysis of mouse SS model

The establishment of SS mouse model employed male C57BL/6J mice. A total of 16 mice were divided into NC group, Model group, AAV-NC group, and AAV-POSTN groups (n = 4 each). Mice were randomly allocated to each group by assigning a random number from 1 to 16 to each mouse. All investigators except the technician who conducted the animal experiments were blinded to the group assignment throughout the study. The SS model was constructed by immobilization of left shoulder joints according to previous protocols (Qi et al., 2025a). In detail, a 4–0 suture needle was passed around the distal end of the humerus and through the scapula of the mouse. The suture was then tightened and knotted to maintain shoulder joint immobilization. At 1-week post-surgery, animals received intra-articular injections of 20 μL PBS or adeno-associated virus (AAV) particles. All animals were housed under a 12-h light-dark cycle with free access to food and water. NC group received sham surgery. Model group received immobilization combined with PBS injection. Those receiving immobilization combined with AAV injection (1 × 10^12^ GC/mL) were designated as AAV-NC group or AAV-POSTN group, respectively. Three weeks postoperatively, all animals were euthanized with overdose CO_2_ and capsules were collected. Histological analysis was performed to assess cell infiltration, capsule thickness and the MMT rate. ROM assessment was performed using a published methodology (Luo et al., 2022), with all measurements conducted by an investigator blinded to group allocation.

### 4D lable-free proteomic analysis

Ten capsule samples (5 for NC group, five for SS group) were snap-frozen. Samples were homogenized in SDT buffer with quartz sand using an automated homogenizer, followed by sequential ultrasonication, boiling, and centrifugation. Then, supernatants were filtered. For each sample, 20 μg proteins underwent denaturation and separation via SDS-PAGE.

Aliquots containing 200 μg proteins were mixed with dithiothreitol (DTT), depleted of low-molecular-weight components, and alkylated with iodoacetamide. Proteins were treated with trypsin at 37 °C overnight. Resulting peptides were desalted and quantified by UV absorbance at 280 nm. NanoElute-based separation was performed on analytical columns (50 °C) with formic acid (FA) loading, eluted via a 90-min linear gradient. Data was analyzed by Spectronaut (Biognosys AG, Switzerland). Significant difference was defined if p value <0.05 and fold change >2.

### High-throughput RNA sequencing and processing

Total RNA was extracted from 12 capsule samples (6 for SS, six for NC) by the VAHTS Universal V6 RNA-seq Kit (Illumina-compatible). cDNA libraries were assessed for concentration and size distribution on an Agilent 4200 Bioanalyzer prior to 150-bp paired-end sequencing, with all procedures strictly adhering to manufacturer protocols. Raw reads were quality-filtered using Seqtk (v1.3) (Kim et al., 2015). Transcript quantification with StringTie (v1.3.3b) incorporated TMM normalization (Robinson and Oshlack, 2010), and differential expression analysis implemented in edgeR (v3.42.0) identified significant differentially expressed genes (DEGs) meeting thresholds of |log_2_FC| > 2 and p < 0.05.

### Data download and identification of shared DEGs

Gene expression profiles for adhesive capsulitis (AC) were retrieved from the Gene Expression Omnibus. The GSE140731 dataset comprising 48 samples (26 cases, 22 controls) was utilized (Kamal et al., 2020). Gene annotations (GENCODE Human Release 40) mapped probe IDs to gene symbols, with maximum expression values retained for genes matched to multiple probes. Differential expression was analyzed using DESeq2 (v1.42.0), defining significant DEGs as those with |log_2_FC| > 2 and p < 0.05. Intersection analysis identified 14 shared DEGs between SS and AC.

### Cell isolation, culture, and intervention

BMDMs were derived from 8-week-old C57BL/6J mice. After euthanasia and surface disinfection, femurs were collected and bone marrow were flushed with PBS. The collected cells were resuspended in high-glucose DMEM medium with 10% fetal bovine serum and 1% penicillin-streptomycin. Then, cells were treated with macrophage colony-stimulating factor (M-CSF, 20 ng/mL) and were maintained at 37 °C under 5% CO_2_ for 3 days. After that, cells were treated with recombinant POSTN (20 ng/mL) for 48 h.

### Hematoxylin and eosin (HE) and Masson’s trichrome staining

Shoulder capsules were fixed, dehydrated, embedded, and then sliced into 8 μm thick sections. After deparaffinization, sections were stained with hematoxylin, and then counterstained with eosin. Microscope (LEICA DM2500) was used for examination and image acquisition.

Masson staining was conducted using a commercial kit (G1006, Servicebio). Stained sections were sealed and images were captured using microscope (LEICA DM2500).

### Immunofluorescence staining

Immunofluorescence staining was performed according to established protocols (Qi et al., 2025b). Sections were treated with antigen retrieval solution (G0142, Servicebio) and blocking buffer (P0102, Beyotime). Next, the sections were incubated with primary antibodies (CD68, DF7518, Affinity; α-SMA, AB7817, Abcam; POSTN, 66491-1-Ig, Proteintech) at 4 °C overnight, and then incubated with secondary antibodies. The nucleus was stained with DAPI.

For cell immunofluorescence staining, BMDMs were fixed and blocked. Then, cells were incubated with corresponding primary and secondary antibodies, followed by nuclear counterstaining with DAPI. Finally, images were captured by fluorescence microscope.

### Western blot (WB)

Proteins from shoulder capsules were extracted. Then, proteins were separated and transferred onto PVDF membranes. After blocking, the membranes were incubated with primary antibodies (Col 1,72026S, Cell Signaling Technology; α-SMA, AB7817, Abcam; POSTN, 66491-1-Ig, Proteintech) and HRP-conjugated secondary antibodies. The blots were visualized by ChemiDoc MP Imaging System (12003154, Bio-Rad).

### Quantitative real-time polymerase chain reaction (qRT-PCR)

Total RNA was isolated from cells using RNA purification kit. The RNA concentration was measured by NanoDrop (ThermoFisher). Then, cDNA was synthesized. The qRT-PCR was then performed using a real-time PCR system (ThermoFisher). The relative mRNA expression levels were quantified using the 2^−ΔΔCT^ method.

### Statistical analysis

All data was analyzed with GraphPad Prism and was presented as mean ± SD. Group comparisons were conducted employing Student’s t-test and one-way ANOVA. P < 0.05 was regarded as statistical significance.

## Results

### Significant fibrosis was observed in the SS capsules

**FIGURE 1:**
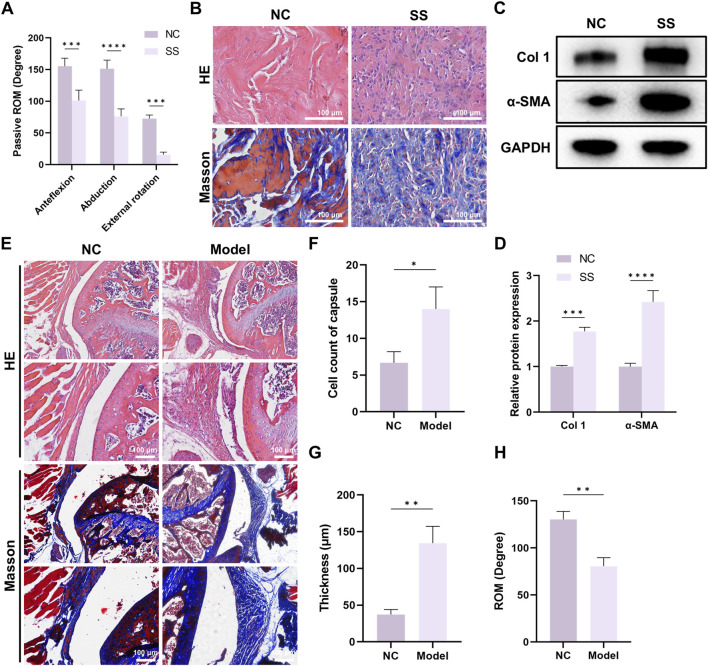
Significant fibrosis was observed in the capsule of SS patients and SS mouse models. **(A)** Passive ROM of patients with or without SS. **(B)** Representative HE and Masson staining images of capsules from patients with or without SS. Bar = 100 μm. **(C,D)** Western blot demonstrated Col one and α-SMA expression in the capsules. **(E)** Representative HE and Masson staining images of capsules from mice with or without SS. Bar = 100 μm. **(F)** Quantification of cell infiltration in the capsules. **(G)** Average thickness of the capsules. **(H)** Passive ROM of the shoulder joint in mice.

Joint capsules from 10 patients were harvested, including five patients with SS (SS group) and five patients without SS (NC group). The baseline characteristics were presented in Table 1. There was no significant difference in basic features between patients with or without SS. ROM assessment showed that the ROM in anteflexion, abduction, and external rotation of patients in SS group was significantly lower than that in NC group (Figure 1A), indicating severe joint stiffness. HE and Masson staining demonstrated that there was increased inflammatory cell infiltration and collagen deposition in the capsule of SS patients (Figure 1B). In addition, WB results showed that Col one and α-SMA expression was significantly elevated in SS group (Figures 1C,D).

**TABLE 1:** Basic characteristics of SS and non-SS patients.

Items	Non-SS	SS	p value
Sex (male:female)	2:3	2:3	>0.999
Age (year)	58.0 ± 3.6	61.8 ± 7.9	0.357
Height (cm)	166.6 ± 6.8	161.6 ± 9.7	0.373
Weight (kg)	69.4 ± 11.9	65.2 ± 14.7	0.632
Duration (month)	3.6 ± 3.3	3.0 ± 1.9	0.732

We further established mouse SS model. The capsules of SS mice also exhibited enhanced cell infiltration, capsule thickening and capsule fibrosis (Figures 1E–G), and ROM was significantly decreased in SS group (Figure 1H). Together, these findings suggested that both SS patients and SS mice developed significant capsule fibrosis, and indicated the successful establishment of mouse SS model.

### MMT occurred in the progression of SS

**FIGURE 2:**
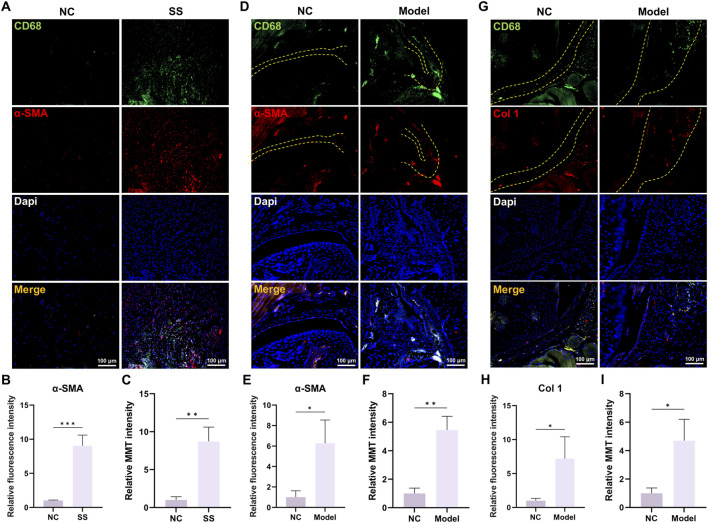
MMT was discovered in the capsules of SS patients and SS mice. **(A,B)** Immunofluorescence staining demonstrated CD68 and α-SMA expression in human capsules. Bar = 100 μm. **(C)** Relative MMT cells intensity. **(D,E)** Immunofluorescence staining demonstrated CD68 and α-SMA expression in mouse capsules. Yellow dashed line indicated the boundary of capsule. Bar = 100 μm. **(F)** Relative MMT cells intensity. **(G,H)** Immunofluorescence staining demonstrated CD68 and Col one expression in mouse capsules. Yellow dashed line indicated the boundary of capsule. Bar = 100 μm. **(I)** Relative MMT cells intensity.

Next, we validated the involvement of MMT in SS. Immunofluorescence staining demonstrated that the expression of macrophage marker CD68 and myofibroblast marker α-SMA was significantly elevated in the capsules of SS patients (Figures 2A,B). Critically, there were more cells that co-expressed CD68 and α-SMA (MMT cells) in SS group (Figure 2C), indicating a greater MMT rate in the SS capsules.

Studies in mouse SS models also corroborated these findings. Immunofluorescence staining showed significant elevation of CD68/α-SMA and CD68/Col one expression in Model group, as well as increased MMT cells (Figures 2D–I). Collectively, these results confirmed the involvement of MMT in the SS progression.

### Multi-omics analysis identified POSTN as a potential pathogenic molecule in SS

**FIGURE 3:**
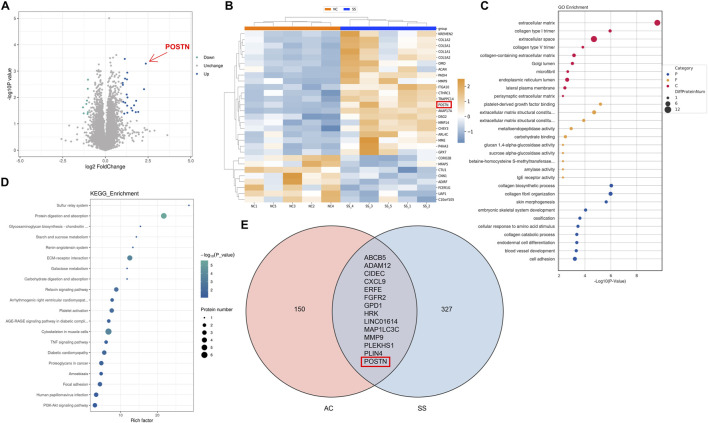
Multi-omics analysis identified POSTN as a potential pathogenic molecule in SS. **(A)** Volcano diagram and **(B)** heatmap showed 29 DEPs with 21 upregulated and eight downregulated. **(C)** GO enrichment analysis and **(D)** KEGG pathway enrichment analysis demonstrated the highly enriched functions and signaling pathways of DEPs. **(E)** Venn diagram demonstrated the intersection of DEGs from transcriptomic analysis.

After confirming the occurrence of MMT in SS progression, we further investigated the potential regulatory factors of MMT. The proteomic analysis on capsule samples of patients was conducted and revealed a total of 29 differentially expressed proteins (DEPs), with 21 upregulated and eight downregulated. Among these, periostin (POSTN) expression increased the most significantly in the SS capsules (Fold change >5.1, p < 0.001) (Figures 3A,B). POSTN is an ECM protein which is known to play a crucial role in promoting cell adhesion, migration, and proliferation. It has been verified to be involved in various fibrotic disorders. This result raised our attention on POSTN. GO and KEGG enrichment analysis demonstrated that the DEPs were highly enriched in terms related to ECM remodeling and cell adhesion (Figures 3C,D), consistent with the pathophysiological changes of SS.

Furthermore, we performed transcriptomic analysis on our collected capsule samples and combined these results with the transcriptomic data of SS capsules from the GEO database for joint analysis. We identified 14 DEGs from both transcriptomic datasets, including POSTN (Figure 3E). These results indicated that POSTN played a critical role in SS progression and might be a potential pathogenic factor of SS.

### POSTN induced MMT in BMDMs

**FIGURE 4:**
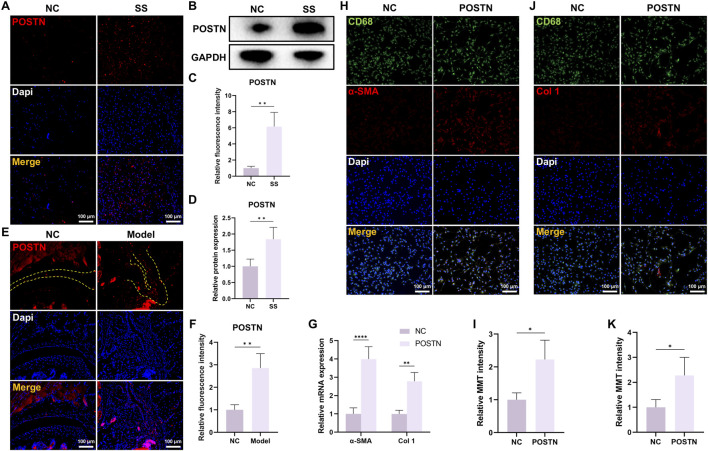
POSTN was highly expressed in the capsules of SS and induced MMT in BMDMs. **(A)** Immunofluorescence staining and **(B)** Western blot showed the POSTN expression in human capsules. Bar = 100 μm. **(C,D)** Quantification of fluorescence intensity and protein expression. **(E,F)** Immunofluorescence staining showed the POSTN expression in mice capsules. Yellow dashed line indicated the boundary of capsule. Bar = 100 μm. **(G)** qRT-PCR showed the relative mRNA expression of α-SMA and Col one in BMDM after different treatments. **(H)** Immunofluorescence staining demonstrated CD68 and α-SMA expression in BMDMs. Bar = 100 μm. **(I)** Relative MMT cells intensity. **(J)** Immunofluorescence staining demonstrated CD68 and Col one expression in BMDMs. Bar = 100 μm. **(K)** Relative MMT cells intensity.

Our previous researches identified POSTN as the potential pathogenic molecule. Then, we verified the POSTN expression within capsules of both SS patients and SS mice. Immunofluorescence staining and WB showed that the expression level of POSTN was significantly upregulated in the capsules of SS patients (Figures 4A–D). Similarly, the POSTN expression in the capsules of SS mice was also elevated (Figures 4E,F). These findings verified the results of multi-omics analysis.

To investigate whether POSTN possessed a regulatory effect on MMT, we isolated BMDMs from mice and stimulated them with POSTN. The qRT-PCR result demonstrated that the mRNA expression levels of α-SMA and Col one were significantly elevated in POSTN-stimulated BMDMs (Figure 4G). Immunofluorescence staining results demonstrated that the expression of myofibroblast marker α-SMA and Col one were significantly increased in BMDMs after POSTN stimulation (Figures 4H–K), indicating that POSTN was an important molecule regulating MMT.

### POSTN knockdown attenuated MMT and capsule fibrosis *in vivo*

**FIGURE 5:**
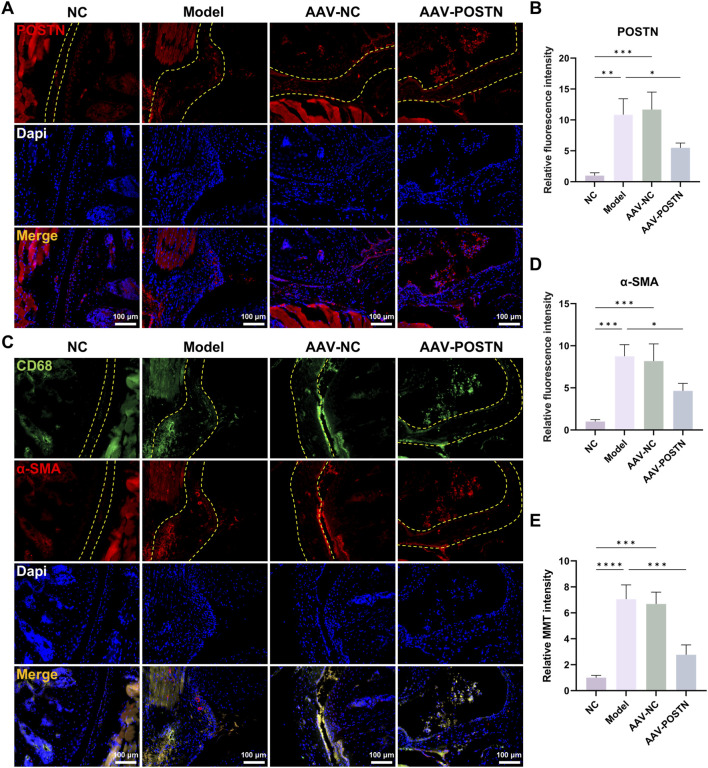
Knockdown of POSTN attenuated MMT *in vivo*. **(A,B)** Immunofluorescence staining showed the POSTN expression in mice capsules. Yellow dashed line indicated the boundary of capsule. Bar = 100 μm. **(C,D)** Immunofluorescence staining demonstrated CD68 and α-SMA expression in mice capsules. Yellow dashed line indicated the boundary of capsule. Bar = 100 μm. **(E)** Relative MMT cells intensity.

Based on our *in vitro* findings that POSTN induced MMT in BMDMs, we further investigated the impact of POSTN inhibition on MMT and capsule fibrosis *in vivo*. We constructed an AAV vector to knockdown POSTN (AAV-POSTN). Then, it was delivered into the shoulder joint of SS mice through intra-articular injection. Immunofluorescence staining demonstrated that the AAV-POSTN significantly downregulated POSTN expression in the SS mice capsule compared to the Model group (Figures 5A,B), indicating the successful knockdown of POSTN *in vivo*.

**FIGURE 6:**
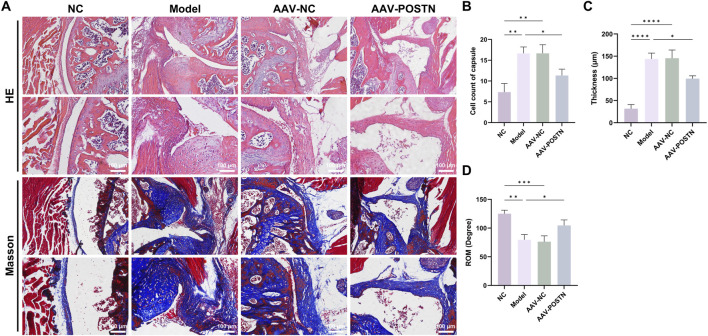
Knockdown of POSTN alleviated capsule fibrosis *in vivo*. **(A)** Representative HE and Masson staining images of capsules from mice with different treatments. Bar = 100 μm. **(B)** Quantification of cell infiltration in the capsules. **(C)** Average thickness of the capsules. **(D)** Passive ROM of the shoulder joint in mice.

Additionally, POSTN knockdown markedly downregulated α-SMA expression and number of MMT cells in the capsules, as shown by immunofluorescence staining (Figures 5C–E). HE and Masson staining demonstrated that the inhibition of POSTN significantly reduced cell infiltration and capsule thickness, as well as restored ROM compared to Model group (Figures 6A–D). No adverse event was observed. Together, these results suggested that POSTN knockdown effectively attenuated MMT and ameliorated capsule fibrosis *in vivo*, indicating that POSTN held the potential to become the therapeutic target for SS.

## Discussion

In this study, joint capsules from patients were collected and the mouse SS model was established. Then, we verified that MMT was involved in the progression of SS. Multi-omics analysis identified POSTN as a potential pathogenic factor, and we verified that POSTN directly induced MMT in BMDMs. Finally, *in vivo* studies proved that targeted suppression of POSTN significantly attenuated MMT and fibrosis within the capsules.

SS is characterized by chronic inflammation and progressive capsule fibrosis which lead to pain and ROM restriction (Ramirez, 2019; Leafbl et al., 2023). The onset of SS is accompanied by the activation of myofibroblasts which excessively secrete collagen into ECM. Thus, inhibiting myofibroblasts activation is crucial for treating SS (Millar et al., 2022; Dias et al., 2005). In this study, we demonstrated that the passive ROM of SS patients were significantly decreased, and the cell infiltration, collagen deposition, and fibrotic proteins expression in SS patients’ capsules were significantly upregulated. Meanwhile, the capsules from mouse SS models also manifested marked fibrosis. These results confirmed the reliability of human capsule samples and the mouse SS model for subsequent experiments.

During the early stage of SS, the joint capsule is infiltrated by macrophages (Dias et al., 2005). In addition to classic M1/M2 polarization, macrophages can transdifferentiate to myofibroblasts to acquire fibrotic features and express α-SMA. This process is called MMT (Li et al., 2024; Yu et al., 2024). The MMT has been identified in various fibrotic diseases. However, the involvement of MMT in SS is still unknown. In this study, we provided the first evidence that MMT occurred in the capsules of both SS patients and SS mice. This finding supported MMT as a novel mechanism underlying the pathogenesis of SS.

POSTN is an ECM protein first identified from a murine osteoblastic cell line in 1993 (Takeshita et al., 1993). POSTN is expressed in diverse tissues and is involved in various processes including hyperplasia, fibrosis, and tumor metastasis (Wu et al., 2024). Previous researches demonstrated that POSTN could activate myofibroblasts and promote ECM deposition in multiple fibrotic diseases (Kotobuki et al., 2014; Kikuchi et al., 2014). Cao et al. reported elevated POSTN expression in idiopathic pulmonary fibrosis and demonstrated that POSTN inhibition significantly reduced fibrotic protein expression in lung fibroblasts (Wu et al., 2024). Similarly, Li et al. confirmed a strong correlation between increased POSTN expression and the development of liver fibrosis (Chen et al., 2020). In the current study, we employed multi-omics analysis of capsule samples and identified POSTN as a potential pathogenic molecule. Subsequent experiments confirmed that the POSTN expression was significantly elevated in the SS capsules. Furthermore, we validated that POSTN directly induced MMT in BMDMs. These findings strongly suggested that POSTN was the key regulator of MMT and could be a promising therapeutic target for SS.

In order to investigate the therapeutic effect of POSTN inhibition on SS, we employed a mouse SS model. This model was established through joint immobilization and was widely employed in SS research (Luo et al., 2022; Qi et al., 2025b; Sun et al., 2022; Yan et al., 2023). The model effectively simulated the pathological features of clinical SS patients, including inflammation, capsule fibrosis and ROM restriction (Qiao et al., 2023; Chen et al., 2021). Since POSTN is widely distributed in connective tissues and participates in critical physiological processes (Izuhara et al., 2019; Sonnenberg-Riethmacher et al., 2021; Jin X. et al., 2024), it is essential to locally suppress POSTN within the capsule to minimize potential adverse effects. In this study, intra-articular injection of AAV-POSTN was conducted for targeted inhibition of POSTN *in vivo*. Histological analysis showed that POSTN knockdown significantly inhibited MMT, ameliorated capsule fibrosis and improved ROM, indicating that POSTN had the potential to become the therapeutic target for SS through MMT inhibition.

The identification of the POSTN-MMT axis provides new insights into the therapeutic intervention for SS. Future development of POSTN-targeted therapies such as intra-articular delivery of POSTN-targeted agents may offer a promising direction for clinical translation. However, there are still some challenges, including the specificity and safety of POSTN inhibition, developing effective delivery systems, and reducing the economic burden on patients. Further researches are essential to improve this POSTN/MMT-targeted therapy.

There are still several limitations. First, the SS model was established using healthy mice only. However, SS is associated with various metabolic disorders, such as diabetes and thyroid disorders (Maund et al., 2012). Thus, future studies should employ disease-specific models to investigate the impact of these disease factors on POSTN-targeted therapy. Second, mice are quadrupedal animals and the biomechanics of their shoulder joints differ from humans. This may introduce potential bias when translating findings from mouse model to human. Third, this study did not investigate the specific mechanisms by which POSTN regulates MMT. Future researches should identify the downstream signaling pathway involved in POSTN-induced MMT regulation.

## Conclusion

This study provided the first evidence that MMT occurred in SS capsules and contributed to fibrogenesis. By conducting multi-omics analysis, we identified POSTN as a key regulatory factor of MMT. *In vitro* experiments demonstrated that POSTN could induce MMT in BMDMs. Additionally, *in vivo* POSTN knockdown significantly inhibited MMT, ameliorated capsule fibrosis, and restored ROM. This study provided a novel insight into the pathogenesis of SS and identified POSTN as a promising therapeutic target for SS.

## Data Availability

The datasets generated and/or analyzed in this study are available from the corresponding author upon reasonable request.
